# Mapping the global research landscape and hotspot of exercise therapy and chronic obstructive pulmonary disease: A bibliometric study based on the web of science database from 2011 to 2020

**DOI:** 10.3389/fphys.2022.947637

**Published:** 2022-08-11

**Authors:** Yu Zhou, Xiaodan Liu, Weibing Wu

**Affiliations:** ^1^ Department of Sports Rehabilitation, Shanghai University of Sport, Shanghai, China; ^2^ School of Rehabilitation Science, Shanghai University of Traditional Chinese Medicine, Shanghai, China

**Keywords:** exercise therapy, chronic obstructive pulmonary disease, knowledge mapping analysis, visualization, bibliometrics

## Abstract

**Background:** The application of exercise therapy (ET) in chronic obstructive pulmonary disease (COPD) is generating increasing clinical efficacy and social-economic value. In this study, research trends, evolutionary processes and hot topics in this field are detailed, as well as predictions of future development directions.

**Methods:** Search for literature in the field of COPD and ET and analyze data to generate knowledge graphs using VOSiewer and CiteSpace software. The time frame for the search was from 2011 to January 2021. Then we extracted full-text key information (such as title, journal category, publication date, author, country and institution, abstract, and keyword) and obtained the co-citation analysis. Use hierarchal clustering analysis software developed by VOSviewer to map common citations, and use Citespace software to plot trend networks.

**Results:** The United States topped the list with 27.91% of the number of articles posted, followed by the UK at 25.44%. Imperial College London was the highest number of article publications in institutions, followed by Maastricht University and the University of Toronto. The Royal Brompton Harefield NHS Foundation Trust was one of many research institutions and currently holds the highest average citations per item (ACI) value, followed by Imperial College London and the University of Leuven. Judging from the number of publications related to ET and COPD, it is mainly published in cell biology, respiratory pulmonary diseases, and rehabilitation experiments study medicine. The European Respiration Journal is the most widely published in this field, followed by the International Journal of Chronic Obstructive Pulmonary Disease and Respiratory Medicine.

**Conclusion:** COPD combined with ET is widely used in clinical practice and is on the rise. A distinctive feature of the field is multidisciplinary integration. Rehabilitation research for COPD involves multidisciplinary collaboration, tissue engineering, and molecular biology mechanism studies to help patients remodel healthy breathing. Multidisciplinary rehabilitation measures provide a solid foundation for advancing clinical efficacy in the field of COPD.

## Graphical Abstract

Exercise therapy in COPD: an emerging topic.
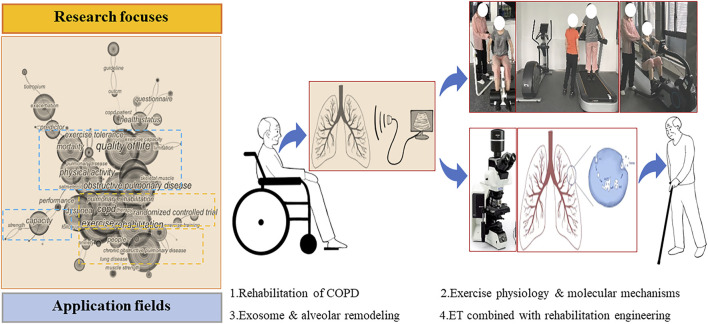

## Introduction

Chronic obstructive pulmonary disease (COPD) is currently defined as “a preventable and treatable disease with some significant intrapulmonary and extrapulmonary inflammatory effects” (Neumeier and Keith, 2020) that may lead to serious consequences such as dyspnea in patients. Pathological manifestations of the lungs are characterized by limited airflow but are not completely reversible (Buist et al., 2007). Airflow restriction usually occurs on an ongoing basis and is associated with an abnormal inflammatory response to lung irritation from toxic particles or harmful gases (Lozano et al., 2012). The common perception of the disease is that it will be one of the major health challenges in the coming decades (GBD 2015 Chronic Respiratory Disease Collaborators, 2017). Prevalence surveys suggested that as many as nearly one in four adults aged 40 years and older worldwide had mild airflow obstruction (Mannino and Buist, 2007)).

Exercise therapy (ET) is a therapeutic form of training that improves the body’s exercise endurance and improves cardiopulmonary function. Many studies have proved that aerobic exercise is an effective way to improve systemic inflammation (Accordini et al., 2020), in particular, has a good effect on improving lung function and exercise ability, and reducing systemic inflammation in COPD patients (Wen et al., 2019). Braz Júnior et al. (2015) found that whole-body vibration (WBV) was effective in improving the 6-min walk distance (6MWD) ability of COPD patients with a good positive modulating effect on the quality of life assessed by the St. George’s Respiratory Questionnaire (SGRQ). Dong J. et al. (2021) study demonstrated that both qigong ET and cycle dynamometer training in COPD patients could improve cardiorespiratory endurance and quality of life, and that cycle dynamometer training played a more effective role in improving the severity of COPD clinical symptoms. Current studies have shown that patients with COPD could improve their exercise ability through short- and medium-to long-term tai chi exercises, and have the potential to improve dyspnea, enhance cardiopulmonary function, and improve the quality of life of COPD patients (Guo et al., 2016; Liu et al., 2021a).

According to the latest studies, ET has achieved a lot of signature progress in the treatment of COPD. As an important means of COPD pulmonary rehabilitation, long-term regular rehabilitation exercises have a good rehabilitation effect in alleviating patients’ breathing difficulties, delaying skeletal muscle atrophy, improving quality of life, and can effectively reduce the level of COPD systemic inflammation. Liu et al. (2021b) found that the oral administration of N-acetylcysteine (NAC) relieves inflammation in COPD patients and could regulate the imbalanced state of T helper 17 cells and regulatory T cells (Th17/Treg). From the perspective of the immunological concept, this result provides new diagnostic and therapeutic means for elderly patients with COPD. Similarly, 6 months of home-based aerobic exercise significantly reduced C-Reactive Protein in serum (CRP serum) and interleukin-8 (IL-8) levels in patients with COPD, and increased skeletal muscle strength and improved exercise capacity; compared with the control group, aerobic exercise groups-maintained serum Tumor Necrosis Factor Alpha (TNF-α) and interleukin-6 (IL-6) levels, suggesting that aerobic exercise could maintain or reduce inflammation levels and slow disease progression (Wang et al., 2014). Bernardi et al. (2015) found that respiratory muscle training improved ventilation patterns and uncoordinated movements of the thoracic cage in patients with COPD, increasing oxygen saturation during endurance training. Abd El-Kader et al. (2016) compared the anti-inflammatory effects of aerobic exercise and resistance training on COPD systemic inflammation. The result showed that both aerobic and resistive exercise could reduce serum TNF-α, interleukin-2 (IL-2), interleukin-4 (IL-4), IL-6, and CRP levels, and the effect of aerobic exercise on the reduction of the above inflammatory factors was significantly better than that of resistive exercise (Abd El-Kader et al., 2016). Based on the brain plasticity developed by the human body’s motor ability, ET has practical clinical significance and cost-effective economic value prospects.

Bibliometrics had the characteristics of bibliology and bibliometrics for quantitatively and qualitatively analysis (Chen, 2006). All included literature was allowed to quantitatively measure the contour distributions as well as study connections and clusters (Chen et al., 2014). Trends in this field of study were described and predicted by summarizing various literature data, including comparing contributions from different countries, institutions, journals, and authors (Chen, 2004). These data analysis techniques have been increasingly used in the development of guidelines and the evaluation of research trends (van Eck and Waltman, 2010). Many researchers in the medical field have adopted this method of literature analysis, such as respiratory medicine research (Waqas et al., 2020), biological signalling molecule research (Liang et al., 2021), genetics research (Alvarez-Peregrina et al., 2021), health information research (Dang et al., 2021), and endocrine disease research (Dong X. et al., 2021).

This study is based on literature data from Exercise Therapy and Chronic obstructive pulmonary disease on the Web of Science website, and the maps of the knowledge graph were done by VOSviewer and CiteSpace. The study presents the latest advance in the field of basic or clinical research in COPD, the evolution of hotspot research, and the prediction of future trends.

## Methods

### Data sources and search strategy

**FIGURE 1:**
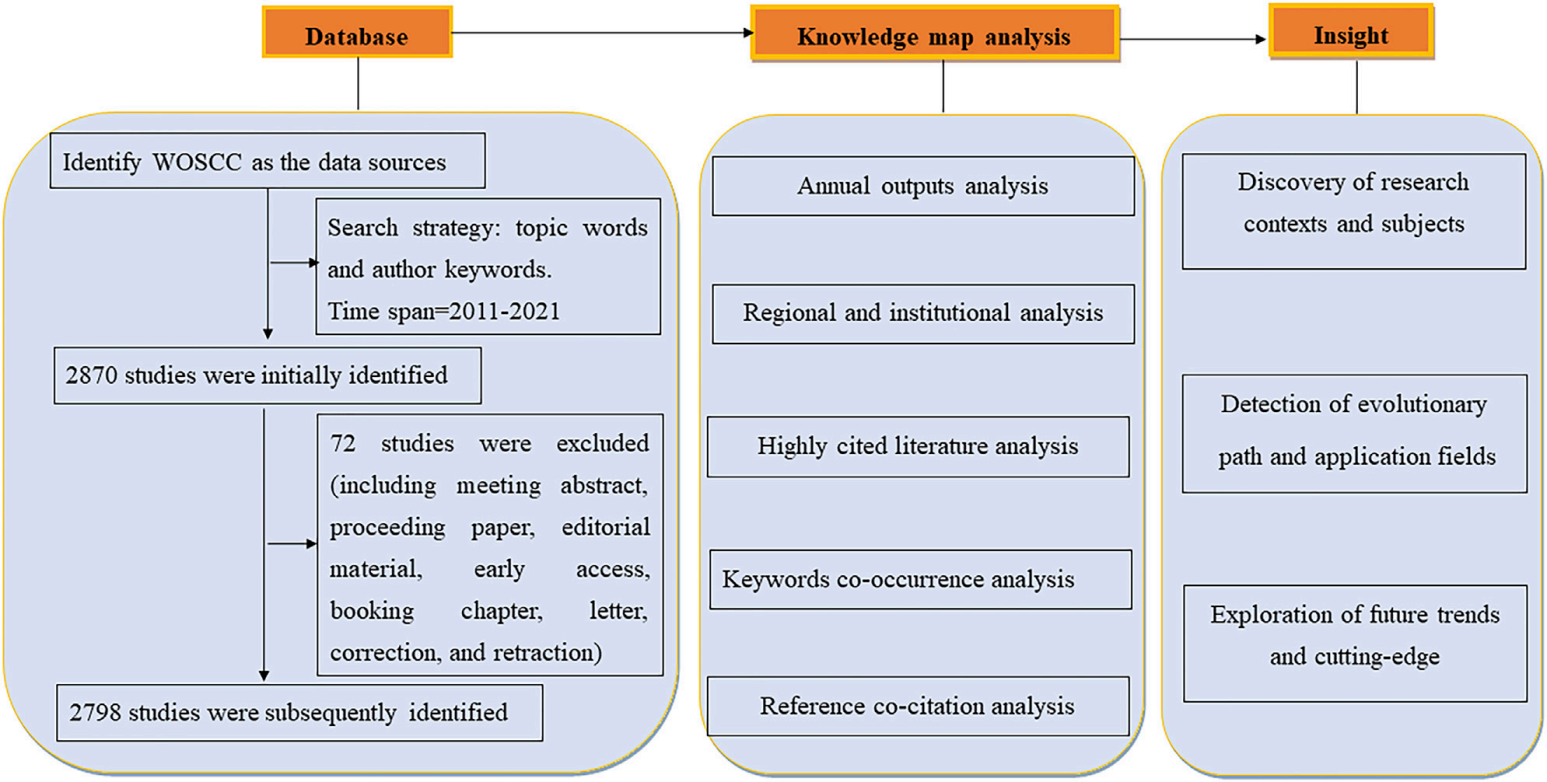
Flowchart of study selection (Preferred Reporting Items for Exercise (ET) therapy and chronic obstructive pulmonary disease (COPD).

We identified the target databases for source literature searches as Science Citation Index Expanded (SCI-EXPANDED) and Social Science Citation Index (SSCI) of the Web of Science Core Database. The search formula was set to TS= (exercise* OR training* OR physical exercise* OR aerobic movement* OR exercise therapy* OR movement therapy* OR kinesiotherapy* OR rehabilitation exercises*) AND TS= (chronic obstructive pulmonary disease* OR COPD* OR chronic airflow obstruction* OR chronic obstructive lung disease*) and the search dates are from 1 January 2011, to 31 December 2020, with a total of 2,870 records searched (Figure 1).

### Inclusion and exclusion criteria

Original articles or commentaries published in peer review on the molecular mechanisms of exercise for COPD were incorporated. The exclusion criteria are 1) meeting summary or errata document; 2) Unpublished articles; 3) Duplicate publications; 4) Irrelevant articles.

### Bibliometrics and visual analysis

2,798 articles were retrieved and imported into VOSviewer and CiteSpace for analysis. Firstly, calculating similarity matrices based on the co-occurrence matrices, CiteSpace is used to focus on discovering and analyzing the evolution of research trends and the relationships between cutting-edge hotspots and their associated knowledge bases (Chen, 2004). CiteSpace was also used to look for the intrinsic connections between different hotspots to obtain keywords related to strong co-occurrence bursts that serve as predictors of research trends to analyze the latest direction. Then, the similarity matrix technique for constructing the map was done by VOSviewer software. The term co-occurrence diagram in VOSviewer included only articles mentioned at least 50 times in the title and abstract and was analysed by binary algorithm counts. VOSviewer constructs maps based on co-occurrence matrices to complete the translation, multidimensional rotation, and panoramic mapping in map construction (van Eck and Waltman, 2010).

## Results

### Distribution of articles by year of publication

**FIGURE 2:**
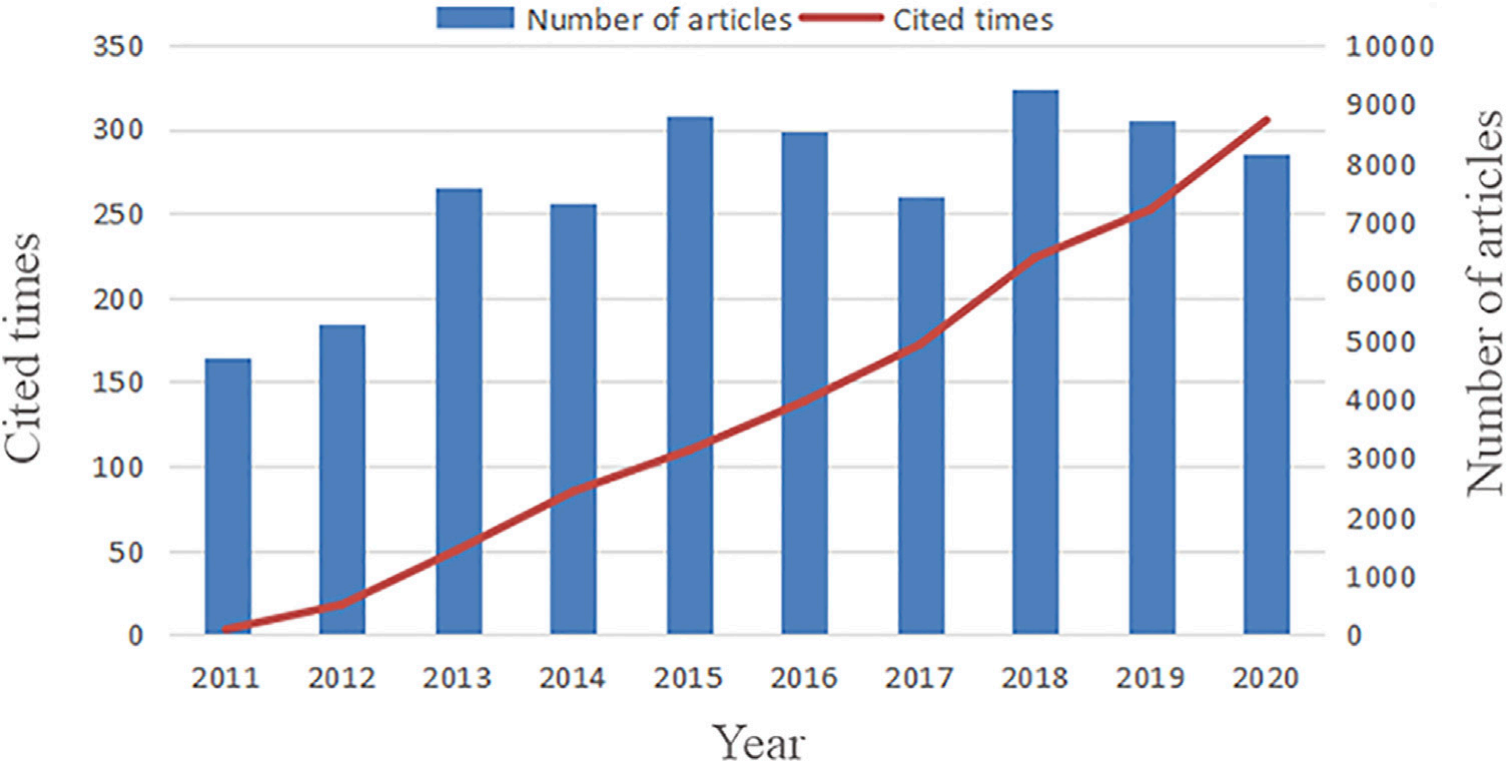
Trends in the growth of publications and the number of cited articles worldwide from 2011 to 2020.

The search date was from 1 January 2011, to 31 December 2020, and a total of 2,798 articles were obtained after deleting 72 non-conforming records. From 2011 to 2020, the number of research papers published in this field showed an overall rapid upward trend (Figure 2), from 5,000 1-year articles to about 10,000 articles a year. During these 10 years, the number of articles peaked in 2018, reaching nearly 10,000 publications a year, and then declined in 2019 and 2020. In terms of citations, the number of citations published between 2011 and 2021 also increased year by year, and in 2020, the number of citations peaked, indicating that more and more researchers are beginning to pay attention to this field.

### Country/region distribution

Table 1 was shown the top two countries in terms of the number of articles published, ranking the United States at 15.26% and the United Kingdom at 13.33%. The total number of articles published by the two countries accounts for one-third of the total, and other countries such as Australia, Canada, and Brazil also accounted for about 10%, which showed that all countries had a strong willingness to study and innovate in this regard. The average citation rate of papers was expressed in average citations per item (ACI). Total link Strength was the total number of co-occurrences of keywords and other keywords in the VOSviewer software (including the number of repeated co-occurrences), the number of nodes indicated the number of connections with the node, the more the total number of connections, the larger the circle. China (42.32), the United States (34.9), and Netherlands (34.07) are the top three countries in ACI value, indicating that their research systems were established earlier and the results of the research were more mature.

**TABLE 1:** Top 10 productive countries.

Rank	Country	Region	Quantity	Percentage (%)	ACI	H-index	Total link strength
1	United States	North America	411	27.91	34.9	81	446
2	England	Western Europe	359	25.44	16.7	49	279
3	Australia	Australia	274	8.47	25.37	43	178
4	Canada	North America	259	6.93	31.10	44	143
5	Brazil	South America	223	6.34	30.46	39	79
6	Netherlands	Western Europe	215	5.85	34.07	39	64
7	Italy	Southern Europe	195	5.85	31.55	38	152
8	Germany	Central Europe	190	5.30	22.43	31	99
9	China	East Asia	180	3.97	42.32	38	77
10	France	Western Europe	155	3.94	31.03	33	57

ACI, average citations per item.

We selected publications published from 2011 to 2021 with a time slice of 1 year and selected the 30 most cited or appeared items from each slice. Selected tree ring history, for example, tree circle size, in node display mode, the size of which represented the number of papers published by a country, author, or institution. The lines between nodes represented cooperation, and the colour of the lines represented clustering. Rings of different sizes in the Atlas indicated frequency, different colours indicated different years, and the outermost purple circle indicated centrality. Nodes with a Fuchsia colour indicated important nodes with strong centrality, while other yellows were highlighted as key nodes affecting the field.

**FIGURE 3:**
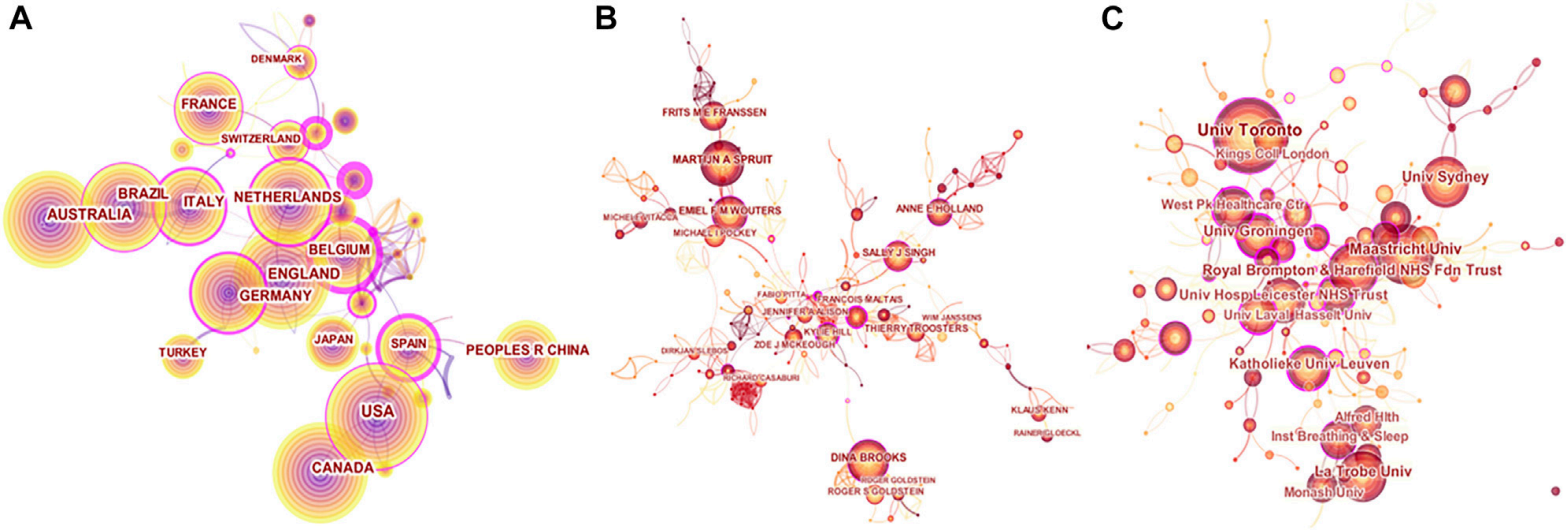
Cooperation map **(A)**. Co-countries **(B)**. Co-authors **(C)**. Co-institutions in the studies of the COPD and Exercise Therapy. Figures 3A–C are used by CiteSpace.

The colour of the circle represents the year of publication, and countries with close cooperation were shown with strong centrality key points of the purple circle. We found that the United States was the country that publishes the most papers and had close exchanges with Spain and Canada. European countries, mainly the United Kingdom, had also published a large number of papers in this field and had close exchanges with each other (Figure 3A). Many countries tended to work with a relatively stable country, generating three major national clusters in the graph, each often containing one or more national core research teams. Many authors tended to work with a relatively stable team of collaborators, so 4 major author clusters were generated in the graph, each of which typically contained two or more core authors (Figure 3B). The chart in Figure 3C was shown the cooperative institutions in this field, many of which had a considerable amount of publications, and maintained a certain degree of cooperation.

### Distribution of authors and research institutions

Table 2 expounded that Spruit, M. A. of Maastricht University in Netherlands published the most articles, followed by Wouters, E. F. of Maastricht University. Three of the top ten writers are from Netherlands.

**TABLE 2:** Top 10 authors in the studies of COPD and exercise therapy.

Rank	Author	Country	Institute	TP	Percentage (%)	H-index
1	Spruit, M. A	Netherlands	Maastricht University	83	3.08	25
2	Wouters, E. F	Netherlands	Maastricht University	61	2.26	23
3	Brooks, D	Canada	University of Toronto	59	2.19	22
4	Franssen, F	Netherlands	Centre of Expertise for Chronic Organ Failure	52	1.93	19
5	Holland, A. E	Australia	Monash University	52	1.93	16
6	Troosters, T	Belgium	Catholic University Leuven	50	1.85	21
7	Singh, S. J	England	University of Leicester	46	1.70	22
8	Hill, K	Australia	Curtin University	45	1.67	16
9	Polkey, M. I	England	Royal Brompton Hospital, Imperial College	42	1.56	20
10	Pitta, F	Belgium	University Hospital Gasthuisberg -University of Leuven	40	1.48	14

TP, total publications, h H-index.

Table 3 was shown that the institution with the most publications in the field of research was the College London with 108 copies, followed by Maastricht University in Netherlands with 95 copies and the University of Toronto in Canada with 87 copies. The contribution of a research institution was expressed in ACI value, and the highest ACI value in 10 years was Royal Brompton Harefield NHS Foundation Trust, with an ACI of 42.38, the second was Imperial College London, with an ACI of 36.25, and the third was Catholic University Leuven of Belgium, with an ACI of 35.28.

**TABLE 3:** Top 10 institutions in the studies of COPD and exercise therapy.

Rank	Institution	Country	Quantity	STC	ACI
1	Imperial College London	United Kingdom	108	3,734	36.25
2	Maastricht University	Netherlands	95	2,153	22.66
3	University of Toronto	Canada	87	2,005	23.05
4	University of Leicester	United Kingdom	78	1,906	24.44
5	Monash University	Australia	75	1,034	13.79
6	Royal Brompton Harefield NHS Foundation Trust	United Kingdom	72	3,051	42.38
7	University Hospitals of Leicester NHS trust	United Kingdom	72	1,791	24.88
8	University of London	United Kingdom	71	2,450	34.51
9	Catholic University Leuven	Belgium	67	2,364	35.28
10	Institute for Breathing Sleeps IBAS	Germany	65	960	14.77

STC, sum of the times cited; ACI, average citations per item.

### Distribution of research disciplines

Table 4 outlined indicators based on the number of papers published to identify the top three disciplines, including Liberatory (56.62%), Medicine general internal (9.87%), and Cardiac cardiovascular systems (8.54%). Other disciplines represented in the literature include Health care sciences services (2.63%), Physiology (2.63%) and other disciplines, the above research was shown that the research carried out in this field was extensive and diverse, mainly around skeletal muscle protein degradation (Balnis et al., 2020), genotype (Silverman, 2020), inflammatory (Baldi et al., 2014; Abd El-Kader et al., 2016), and other related studies have been carried out in multiple disciplines.

**TABLE 4:** The top 20 subject categories in the studies of the COPD and exercise therapy.

Rank	Quantity	WOS categories	Percentage (%)	Rank	Quantity	WOS categories	Percentage (%)
1	1,525	Respiratory	56.62	11	46	Public environmental occupational health	1.70
2	266	Medicine general internal	9.87	12	44	orthopaedics	1.63
3	230	Cardiac cardiovascular systems	8.54	13	43	Geriatrics gerontology	1.59
4	224	Rehabilitation	8.31	14	43	Integrative complementary medicine	1.59
5	187	Critical care medicine	6.94	15	43	nursing	1.59
6	107	Sport sciences	3.97	16	31	Medical informatics	1.15
7	85	Medicine research experimental	3.15	17	31	Nutrition dietetics	1.15
8	71	Health care sciences services	2.63	18	31	surgery	1.15
9	71	Physiology	2.63	19	28	Primary health care	1.04
10	63	Pharmacology pharmacy	2.33	20	25	Multidisciplinary sciences	0.92

Table 5 was shown the ranking of journals with the largest number of articles published, the European respiration journal had the largest number of articles with 235 articles, the second most published was the international journal of chronic obstructive pulmonary disease with 189 articles, and Respiratory Medicine ranked third with 91 articles. The COPD: journal of chronic obstructive pulmonary disease, Respiratory care, and Respirology followed by 88, 82, and 82 articles. The contribution of a journal was expressed in ACI value, and the highest ACI value in 10 years was The American Journal of respiratory and critical care medicine (86.31) and BMJ open (86.31), followed by the Cochrane database of systematic reviews (71.49), Thorax (34.11), and BMJ open (86.31). Thorax (Chest).

**TABLE 5:** Top 15 journals in the studies of COPD and exercise therapy.

Rank	Journal title	Quantity	ACI
1	European respiration journal	235	11.54
2	International journal of chronic obstructive pulmonary disease	189	15.84
3	Respiratory medicine	91	22.15
4	COPD: journal of chronic obstructive pulmonary disease	88	11.48
5	Respiratory care	82	12.46
6	Respirology	82	13.77
7	Journal of cardiopulmonary rehabilitation and prevention	75	11.91
8	Chronic respiratory disease	59	15.83
9	Respiration	54	20.69
10	Chest	46	31.30
11	American journal of respiratory and critical care medicine	45	86.31
12	BMC pulmonary medicine	45	16.09
13	Cochrane database of systematic reviews	41	71.49
14	Thorax	35	34.11
15	BMJ Open	27	86.31

ACI, average citations per item.

### Highly cited literature analysis

Table 6 outlined the article “An Official American Thoracic Society/European Respiratory Society Statement: Key Concepts and Advances in Pulmonary Rehabilitation” (Spruit et al., 2013) was the most cited. Spruit et al. (2013) published an official statement on advanced concepts and progressions in the functional recovery of pulmonary rehabilitation through the American Thoracic Society (ATS)/European Respiratory Society (ERS). The important role of respiratory rehabilitation in the precise management of chronic diseases was highlighted in this statement, and the role of healthy motor behaviour changes in optimizing and maintaining patient outcomes was further discussed. According to this Practice Guideline, pulmonary rehabilitation plays an important role in promoting self-efficacy and optimizing healthy behaviour change. To ensure that exercise training is effective, cardiorespiratory endurance, strength, and/or flexibility are improved by maximizing aerobic capacity and muscle strength through endurance training, interval training, resistance training, respiratory muscle training, and different training modes based on the individual needs of COPD patients.

**TABLE 6:** Top 15 co-cited articles, cited authors and cited references.

Rank	Title	Journal	Type	Authors	Y	C	In	CN
1	An Official American Thoracic Society/European Respiratory Society Statement: Key Concepts and Advances in Pulmonary Rehabilitation	American Journal of Respiratory and Critical Care Medicine	Article	Spruit, M. A., Sally J. et al	2013	1,540	1	1
2	Exercise as medicine - evidence for prescribing exercise as therapy in 26 different chronic diseases	Scandinavian Journal of Medicine & Science in Sports	Article	Pedersen, B. K., Saltin, B	2015	838	1	1
3	Pulmonary rehabilitation for chronic obstructive pulmonary disease	Cochrane Database of Systematic Reviews	Review	McCarthy B, Casey D. et al	2015	710	2	2
4	Diagnosis and Management of Stable Chronic Obstructive Pulmonary Disease: A Clinical Practice Guideline Update from the American College of Physicians, American College of Chest Physicians, American Thoracic Society, and European Respiratory Society	Annals of Internal Medicine	Article	Qaseem, Amir. et al	2011	668	1	1
5	Muscle wasting in disease: molecular mechanisms and promising therapies	Nature Reviews Drug Discovery	Review	Cohen, Nathan. et al	2015	475	3	3
6	An Official American Thoracic Society/European Respiratory Society Statement: Update on Limb Muscle Dysfunction in Chronic Obstructive Pulmonary Disease Executive Summary	American Journal of Respiratory and Critical Care Medicine	Articles	Maltais, Francois. et al	2014	400	1	1
7	Self-management for patients with chronic obstructive pulmonary disease	Cochrane Database of Systematic Reviews	Review	Zwerink, Marlies. et al	2014	331	6	2
8	Pulmonary Hypertension in Chronic Lung Diseases	Journal of the American College of Cardiology	Review	Seeger, Werner, et al	2013	325	17	8
9	The minimum clinically important difference for the COPD Assessment Test: a prospective analysis	Biomaterials	Article	Kon, Samantha S. C.et al	2011	289	3	1
10	Pharmacology and Therapeutics of Bronchodilators	Pharmacological Reviews	Review	Cazzola, Mario et al	2012	281	4	2
11	An Official American Thoracic Society/European Respiratory Society Policy Statement: Enhancing Implementation, Use, and Delivery of Pulmonary Rehabilitation	American Journal of Respiratory and Critical Care Medicine	Article	Rochester, Carolyn L.et al	2015	259	7	6
12	An official European Respiratory Society statement on physical activity in COPD	European Respiratory Journal	Article	Watz, Henrik et al.	2014	255	28	8
13	Endobronchial Valves for Emphysema without Interlobar Collateral Ventilation	New England Journal of Medicine	Article	Klooster, Karin et al.	2015	243	3	1
14	The minimal important difference in exercise tests in severe COPD	European Respiratory Journal	Article	Puhan, M. A. et al	2011	240	8	3
15	Pulmonary rehabilitation following exacerbations of chronic obstructive pulmonary disease	Cochrane Database of Systematic Reviews	Review	Puhan, M. A. et al	2016	225	4	4

Y, year; C,citations; IN, institute number; CN, country number.

The second most cited article was “Exercise as medicine—evidence for prescribing exercise as therapy in 26 different chronic diseases” (Pedersen and Saltin, 2015). Pedersen and Saltin (2015) collected the latest evidence-based medical evidence on exercise as a treatment for 26 different chronic lung diseases (such as chronic obstructive pulmonary disease, asthma, cystic fibrosis, and so on). This article proposed different chronic diseases and the effects of ET on the symptoms associated with related diseases, discussed possible mechanisms of action and updated with the best recommendations for the optimal type and dosage of exercise prescriptions. By summarizing the results of evidence-based physical exercise, this Statement found modest evidence of a significant increase in leg muscle strength suggesting that a combination of resistance and endurance training may not improve lung function in patients with COPD. But helped counteract protein degradation in COPD patients (Petersen et al., 2008), increasing cardiopulmonary fitness through effects on muscles and the heart. Optimal exercise prescribing was the ability to achieve gait, balance and breathing function through personalized supervision.

A third frequently cited article was “Pulmonary rehabilitation for chronic obstructive pulmonary disease” (McCarthy et al., 2015). McCarthy et al. (2015) explained that pulmonary rehabilitation, also known as respiratory rehabilitation, can relieve difficulty breathing and physical fatigue, regulating the emotional stress of patients with repeated illnesses. Existing findings strongly supported the need for pulmonary rehabilitation for severe COPD, including at least 4 weeks of exercise training, and in addition to a 6MWD test (a measure of functional exercise) (Puhan et al., 2011), there have been clinical and statistically significant improvements in health-related quality of life assessments such as dyspnea, fatigue, and emotional function (Bolton et al., 2013). These measurements were moderate and can help patients reestablish healthy breathing. The rehabilitation criteria recommended by the Cochrane Collaboration were the evaluation system for routine pulmonary function recovery. It was used to maximize the respiratory quality and life engagement and social value for COPD patients and was an important part of COPD management. This Cochrane Review/meta-analysis review clarified that lung function rehabilitation can improve the health-related quality of life in people with COPD, and future studies should further clarify the ideal cycle of rehabilitation programs, the intensity of training required, and the duration of rehabilitation outcomes. The above articles reflected the economic value and clinical efficacy feasibility of exercise therapy.

Depending on the type of literature, the most frequently cited literature was involved in six reviews and nine monographs. Judging from the publication dates of highly cited documents, 2013–2015 was the most cited, followed by 2011–2012. These time nodes can be seen as two important phases in the development of the study area. According to the number of co-institutions and countries/regions, ten articles dealt with more than three co-institutions and nine articles were related to the participation and cooperation of at least two countries.

### The network of keywords co-citation

Keywords highlight the article’s fundamental content, and you can uncover research frontiers that were continually updating and changing in relevant knowledge fields by skimming through them quickly. In contrast, chronic obstructive pulmonary disease and exercise, the first 20 keywords with high frequency and centrality were listed in Table 7. The keywords cited with high frequency were rehabilitation (745), quality of life (555), lung function (506), dyspnea (338), and motor capacity (331). ET had significant clinical significance in relieving dyspnea (Laughlin and Roseguini, 2008) and fatiguing (Beaumont et al., 2018) in pulmonary rehabilitation, further improving emotional function (Lindenthaler et al., 2018) and enhancing individual confidence and control over their condition. Exercise therapy for prolonged (>6 months), which included aerobic exercise (Reis et al., 2013), endurance exercise (Iepsen et al., 2015), and inspiratory muscle training (Pleguezuelos et al., 2016), had been shown to help improve dyspnea, autonomous impedance capability, and life pleasure and reduced risk factors during acute exacerbations in COPD patients.

**TABLE 7:** The top 20 keywords in the studies of the COPD and exercise therapy.

Rank	Keywords	Occurrences	Total link strength	Rank	Keywords	Occurrences	Total link strength
1	COPD	1,203	8,654	11	Mortality	296	2,137
2	Exercise	764	6,735	12	Program	227	2006
3	Rehabilitation	745	4,567	13	Management	182	1904
4	Obstructive pulmonary disease	698	4,556	14	Exacerbation	172	1743
5	Chronic obstructive pulmonary disease	576	4,487	15	Capacity	170	1872
6	Quality of life	555	4,132	16	Health status	164	1,685
7	Pulmonary activity	506	3,908	17	Therapy	145	1,632
8	Disease	382	3,387	18	Randomized controlled trial	139	1,504
9	Dyspnea	338	2,874	19	Predictor	115	1,489
10	Exercise capacity	331	2,309	20	Exercise tolerance	115	1,297

**FIGURE 4:**
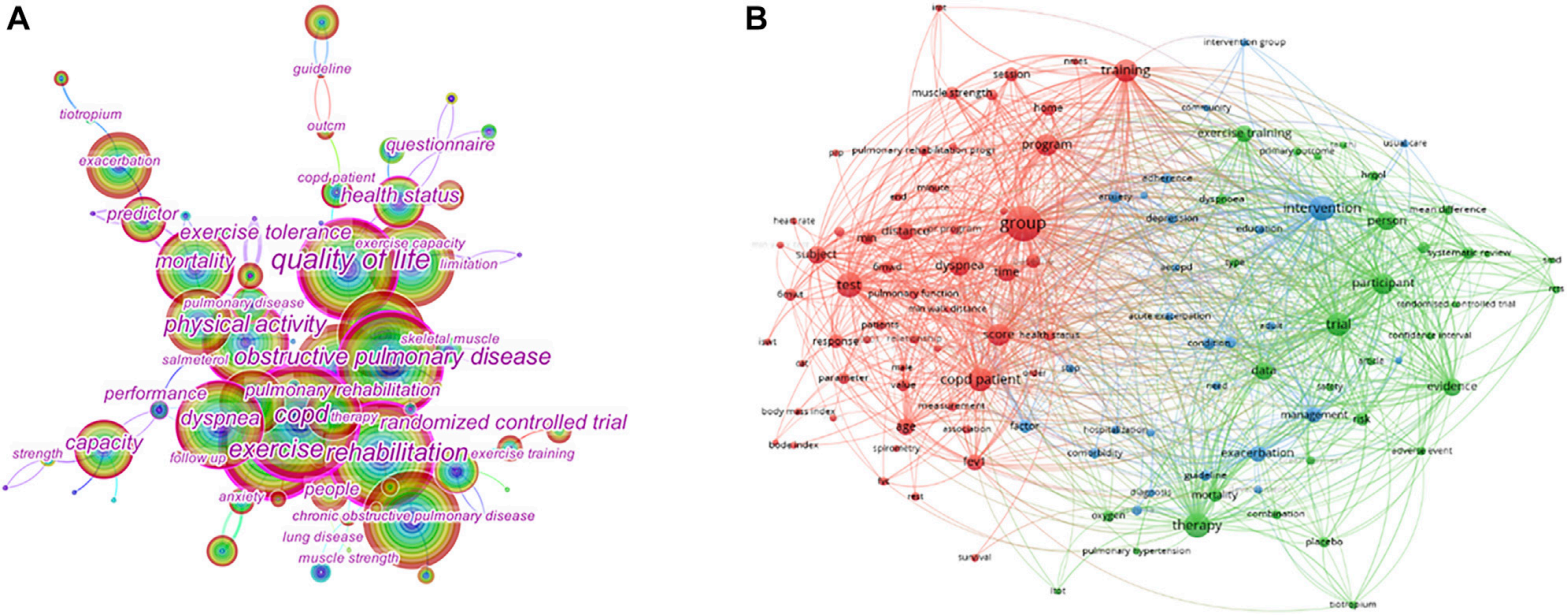
Map of keywords **(A)**. Co-occurring keyword **(B)**. Keyword clustering in the studies of the COPD and Exercise Therapy. Figure 4A is used by Citespace. Figure 4B is used by VOSviewer.

Keyword co-citation and clustering keywords are high-level roundups. High-frequency, highly centralized keywords often reflect research hotspots in the field. We analyzed publications within 1 year, as well as the top 30 levels of publications with the most citations or the most occurrences in each period. The 132 nodes and 987 links consisted of a combined co-occurrence keyword network (Figure 4A). Figure 4B was shown the use of VOSviewer software and presents a keyword co-occurrence network diagram. The more closely connected nodes, the more highly appeared frequency of the two keywords together. The four main research directions were represented by the four clusters of keywords in this field. Red had the largest area, green second, then blue third, and yellow can almost be negligible.

The red clusters consisted mainly of studies related to chronic obstructive pulmonary disease. On the one hand, it included conditions such as dyspnea, Bode index, and 6MWD, which were directly related to the disease. On the other hand, the exploitation of the potential mechanisms of cell transplantation, differentiation, proliferation, mitochondrial dysfunction, inflammation, and apoptosis helped to clarify the practical significance of COPD patients, tests, and subjects. To improve the efficacy of muscle cell transplantation, differentiation and proliferation, regulators of the first three sections (Agustí et al., 2012; Chaillou and Lanner, 2016; Britto et al., 2020; van Doorslaer de Ten Ryen et al., 2021) by drugs (Doucet et al., 2007; van Helvoort et al., 2007; Fiorentino et al., 2020), and tracking tools (Iepsen et al., 2016; Catteau et al., 2021) implemented. The mechanism of the movement was illustrated by the antioxidant, anti-inflammatory, and anti-apoptosis of the latter parts. Mechanisms included exercise-induced production and absorption of glucose in patients with COPD (Franssen et al., 2011), and the action of drugs such as β receptor agonists bronchodilators (Haarmann et al., 2015), metformin (Wu et al., 2021), trypsin-like serine protease inhibitors (Liang and Bowen, 2016), and so on.

The green clusters were mainly composed of research related to sports rehabilitation. On the one hand, several commonly used research methods are described, such as SMD, placebo, and RCTs, which were often used in medical control research. On the other hand, it described the rehabilitation-related situations of training, exercise training, management, and so on. Supplementation with high-intensity interval training (HIIT) in COPD patients compared with placebo groups corrected muscle disorders and improved moderate airflow obstruction and athletic performance (van de Bool et al., 2017). Paneroni et al. (2017) systematically evaluated the effectiveness of ET in patients with very severe COPD, and the meta-analysis results showed that ET could effectively improve exercise endurance, and more randomized controlled trials (RCTs) were needed to further clarify the basis for the scientific formulation of exercise prescriptions. Kerti et al. (2018) studied the degree of contribution of lung exercise training and its evaluation criteria in patients with COPD, and the results showed that the level of exercise tolerance for pulmonary rehabilitation depends on the combined effects of exercise capacity, improvement of inspiratory lung capacity, metabolism and respiratory muscle function. The use of metronomes to set the equation of exercise training intensity improved the respiratory patterns and short-term resistance training of COPD patients during exercise and helped provide supervised exercise parameters for family lung rehabilitation (Robles et al., 2017; Bernardi et al., 2018). COPD self-management improves patient engagement and the sustainability of athletic training through supervised collaboration with physicians and other medical rehabilitation personnel (Bourbeau et al., 2015).

The blue clusters were concentrated in other special cases. Words such as intervention, exacerbation, anxiety, and so forth were being studied for the possible negative effects of COPD on sports disorders. Skeletal muscle dysfunction in patients with COPD occurred not only in peripheral skeletal muscles but also affected the major respiratory diaphragm (Gea et al., 2013). The cause of skeletal muscle dysfunction was related to systemic inflammation, which can lead to changes in skeletal muscle tissue structure and decreased contractility by influencing skeletal muscle protein synthesis and catabolism (Barreiro and Gea, 2016) and apoptosis of skeletal muscle cells (Barreiro et al., 2015). Aerobic exercise can improve the inflammatory response in the body, thereby effectively improving body function (Van Helvoort et al., 2006). Moderate- and high-intensity aerobic exercise can significantly improve the structure and function of skeletal muscles in patients with COPD (Silva et al., 2018). However, there have been relatively few studies on the mechanism of systemic inflammation of skeletal muscle dysfunction in patients with exercise intervention COPD.

### Joint evolutionary path

**FIGURE 5:**
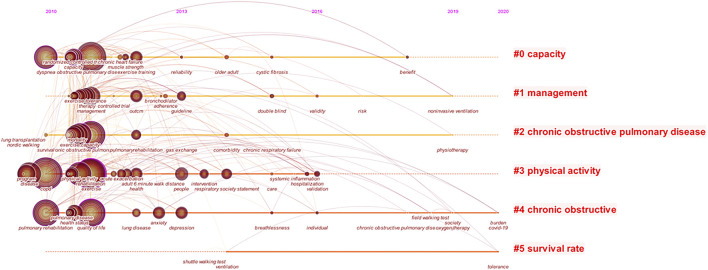
Evolutionary path in the studies of the COPD and Exercise Therapy. Figure 5 is used by Citespace.

In Figure 5, the year corresponding to the dataset analysis indicated that the keyword appeared in the first year. The contact path between COPD and exercise rehabilitation was presented through the transition nodes. From 2011 to 2013, research in the field began focusing on dyspnea, obstructive, mortality, and physiological activity. In 2013–2015, reliability, guidelines, interventions, depression, and breathlessness received increasing attention in the field. The scientific effectiveness of medical research and the prevention and control of safety risks had been a high degree of emphasis from 2016 to 2017, with a new focus from 2018 to 2020 on benefits, safe walking tests and exercise tolerance.

### Research hotspots and cutting-edge analysis

In Table 8, the blue lines presented the timeline, and the red bands represented burst detection, which indicated the start year and duration of the different burst words. In addition, the research-meaningful keywords we were interested in reflect the evolutionary trends in the field. Validity was shown as the strongest explosive intensity, followed by Airflow obstructive, Care, and Hyperinflation. Terms such as Airflow obstructive, Hyperinflation, and Salmeterol began to emerge in 2011, but lasted shorter, ending around 2013. Chronic heart failure, Limitation, and Survival terms were popular between 2014 and 2016. Validation was currently at the forefront of this field of research and is in an explosive stage.

**TABLE 8:** Top 15 keywords with the strongest citation bursts.

Keywords	Strength	Begin	End	2011–2020
Airflow obstruction	9.99	2011	2013	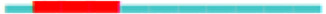
hyperinflation	8.48	2011	2014	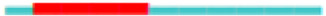
salmeterol	7.11	2011	2012	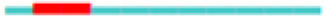
body mass index	6.56	2011	2012	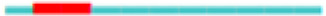
dynamic hyperinflation	5.93	2011	2013	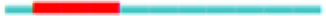
randomized trial	5.9	2011	2013	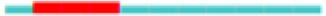
life	5.98	2013	2015	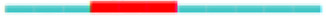
Chronic heart failure	8.26	2014	2016	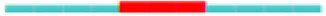
survival	7.95	2014	2015	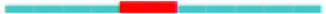
limitation	5.84	2014	2015	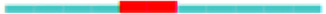
care	9.25	2015	2016	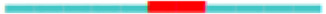
distance	6.35	2015	2016	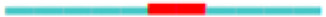
validity	11.31	2016	2017	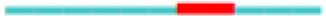
comorbidity	8.09	2016	2017	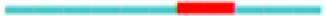
validation	10.46	2018	2020	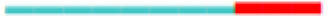

## Discussion

### Summary of findings

This study compiled relevant exercise therapy literature in the field of COPD from 2011 to 2020 and conducted a bibliometric analysis using Citespace and VOSviewer software. The study mainly evaluated the region and publication time of the article, the current research hotspots of COPD combined exercise therapy, and the author’s contribution. Keyword co-occurrence analysis is the hotspot we used to assess and determine researchers’ concerns during each period to uncover core topics in the context of clinical research. Multidisciplinary integration such as alveolar remodelling and rehabilitation engineering is a new trend in our identified research in the field of COPD.

### Rehabilitation of COPD

The affordability of exercise therapy will be well demonstrated in future COPD application treatment; in addition, there are certain risks and limitations, as well as the exercise mode used, exercise capacity, exercise status, exercise pathway, exercise site and intensity, etc., and the treatment effect also varies from person to person. Indications for exercise therapy and patient acceptance of the possible risks that may occur have led to research controversial (Østergaard et al., 2018). For instance, Su et al. (2018) proposed that COPD patients weakened lung ventilation function and decreased oxygen uptake, which led to hypoxemia in organs such as skeletal muscle, which reduced the patient’s ability to exercise and developed waste muscle atrophy. Given the exercise risks and limitations of exercise intensity in the study, the researchers were cautious when conducting their research work. According to a meta-analysis of RCTs of physical exercise intensity, it was confirmed that HIIT and continuous ET had a similar effect on functional capacity and cardiovascular variables in patients with COPD, indicating the clinical effectiveness of ET for pulmonary rehabilitation (Adolfo et al., 2019). Liao et al. (2015) systematically evaluated the safety of resistance training in 750 patients with advanced COPD, and there was evidence that resistance training combined with endurance training significantly improved skeletal muscle strength, improved the total score of the SGRQ, and did not have adverse events under the intervention of scientific resistance training exercise. Inspiratory muscle training (IMT) in patients with COPD relieved excessive activation of the diaphragm and reduced dyspnea during exercise (Langer et al., 2018). A systematic review also found that expiratory muscle training (EMT) combined with IMT in patients with severe to very severe COPD showed that both were effective in improving respiratory muscle strength, but there was no significant difference between the 6WMD and dyspnea (Neves et al., 2014; Xu et al., 2018).

### Exercise physiology and molecular mechanisms

First of all, over the past decade, in Europe and the United States, East Asian countries developed more interest in studying molecular mechanisms in this field. European and American countries have explored the initial research plan and gradually improved their research ideas to achieve clinical utility. In particular, both Imperial College London and the University of Toronto started early and have obtained some high-quality scientific clinical outcomes, including breathlessness rehabilitation (Man et al., 2016) and outcome measures combined ET (Jones et al., 2019), and the effects of post-rehabilitation exercise based on community (Butler et al., 2020). The researchers also explored the pulmonary protection mechanisms of COPD-related quadriceps muscle dysfunction, including sex differences (Sharanya et al., 2019), utilization of non-invasive imaging tools (Rozenberg et al., 2017), handgrip strength reflection (Fonseca et al., 2021), the clustering physiological responses to arm activity (Lima et al., 2016), and the physical activity during moderate (out-patient managed) (Alahmari et al., 2016). Monash University and the University of Bern studied the effects of peripheral muscle training (Bisca et al., 2017), the WBV intervention (Furness et al., 2012), and physical activity significantly correlated with quadriceps strength (Osthoff et al., 2013) on skeletal muscle dysfunction, exercise tolerance and functional performance. Italy’s studies found that respiratory rehabilitation interventions in COPD patients could reduce the inflammatory cytokine IL-6, and improve lung capacity and 6MWD ability (Baldi et al., 2014). In addition, Belgium research found that motor muscle work during HIIT could impair blood flow perfusion of the diaphragm and increase the feeling of dyspnea (Louvaris et al., 2021). Therefore, the United Kingdom’s cooperation with the United States research showed COPD patients needed to be able to achieve noninvasive monitoring of blood flow in the respiratory tract and motor muscles during exercise (Vogiatzis et al., 2020), and both German and Belgian studies have been validated to show that the near-infrared spectrum could be used to assess the respiratory and muscle blood flow index and prevent hyperpnea and muscle perfusion during exercise (Habazettl et al., 2010; Louvaris et al., 2018).

East Asian countries have focused on the dialectical treatment of the holistic view of COPD through the qigong and tai chi of traditional Chinese exercise therapy (TCET) for nearly 10 years (Guo et al., 2016; Dong J. et al., 2021). Among the many scientific research institutions that have made more leading achievements are Guangzhou Medical University, the Royal Brompton and Harefield and other cooperation institutions, such as NHS Foundation Trust and Imperial College. The study explored the physiological response of stable COPD to tai chi was similar to treadmill training, which could improve respiratory rate and diaphragm dynamic hyperinflation (Qiu et al., 2016). Scholars have also explored improvements in lung function and diaphragm thickness through respiratory muscle training (RMT) (Xu et al., 2018; Cheng et al., 2022). RMT devices combined with respiratory techniques could improve respiratory muscle strength, reduce TNF-α and IL-6, and have a significantly improved effect on the plasma level of oxidative stress marker-total antioxidant capacity (TAC) (Leelarungrayub et al., 2018).

### Exercise therapy combined with rehabilitation engineering

Under the premise of multidisciplinary cross-intersection, the development of ET combined with rehabilitation engineering has received great impetus and technical support. The dynamic rehabilitation process of COPD is mainly studied through the literature in the field of computer informatics (McCabe et al., 2017) to further elaborate the mechanism of the intervention effect of exercise therapy. Chronic obstructive pulmonary disease systematically studies the related pulmonary disease of clinical rehabilitation technique and basic laboratory machines, involving clinical rehabilitation engineering, intelligent medical engineering, sport sciences and other disciplines, reflecting that one of the characteristics of the combination of exercise therapy and rehabilitation engineering is the integration and development of disciplines.

Rehabilitation engineering for noninvasive monitoring and widespread applications of wearable materials have increasingly become hot topics of concern for COPD researchers. For example, taking the initiative to participate in video games (Simmich et al., 2019), the active use of resistance breath muscle training devices (Włodarczyk and Barinow-Wojewódzki, 2015; Schaper-Magalhães et al., 2017; Daynes et al., 2018), wearable smart vests (Naranjo-Hernández et al., 2018) and a smartphone oximeter with a fingertip probe (Chan et al., 2019) could effectively monitor the respiratory rate of COPD patients. In addition, using interdisciplinary digital mobility results enabled scientific assessment of the clinical utility of physical activity (Polhemus et al., 2021) and remote rehabilitation systems combined with wearable equipment could help COPD patients to achieve better sports performance and healthy quality of life (Tey et al., 2017). The cross-development of disciplines in different fields will help to break through the technical difficulties that limit the development of a certain field.

### Exosome and alveolar remodelling

Exosome and alveolar remodelling research, and tissue engineering research are hot and clinical efficacy areas in this field that need to be verified. Among the bibliometric results, we found that COPD was associated with exosome and alveolar remodelling in the research hotspots, and exosomes played an important role in the pathogenesis of COPD by regulating chronic inflammation (Tan et al., 2017; Sundar et al., 2019a), mitochondrial dysfunction (Lerner et al., 2016), emphysema (Klooster et al., 2015) and cystic fibrosis (Pedersen et al., 2015), and oxidative stress (Sundar et al., 2013). Exosomes are small lipid-binding vesicles found in lung tissue and bronchoalveolar lavage fluid (BALF) (Kaur et al., 2021). Exosomes act as potential biomarkers to promote cell-to-cell communication under normal and diseased conditions (Sundar et al., 2019b). Patients with COPD with small airway obstruction led to pulmonary ventilation dysfunction due to complex and dynamic hyperinflation alveolar pathological remodelling process. The expansion of the alveoli and worsening of contractile and diastolic function are associated with adaptive changes that occur at different levels, respectively by genetic selection, molecular biology, and cellular interstitial levels.

### Limitations

Our comprehensive bibliometric research has some limitations, and for high-quality bibliometric analysis, the Web of Science database is searched by default, thus excluding non-SCI journals such as BMJ Open Respiratory Research, which may have many publications related to a variety of chronic pulmonary diseases. Furthermore, our group have not yet collected a more detailed analysis of study subjects suffering from multiple diseases in exercise therapy, such as the prevalence of exercise therapy and the burden of non-pharmacological treatment of multiple diseases in each region or centre where the sample had studied. To be inspired by the research program, our team recommended a systematic review of home exercise therapy and COPD topics, covering more databases of home exercise therapy and online therapists guiding lung rehabilitation. Secondary databases require global multi-scope research analysis and preliminary longitudinal cohort scheme design. Under the impact of the COVID-19 pandemic, COPD patients could choose between hospital outpatient sports training or home sports training. A meta-analysis conducted by Wuytack et al. (2018) yielded results that exercise training programs could help COPD patients improve lung respiratory symptoms, health-related quality of life, and exercise capacity, with low and moderate evidence that exercise training was equally effective whether in hospitals or at home.

## Conclusion and future perspective

This research analyzed the research hotspots and frontiers of exercise therapy on COPD through VOSviewer and CiteSpace. The results showed that exercise improves the efficacy of skeletal muscle cell transplantation, differentiation and proliferation of COPD, and exercise antioxidant, anti-inflammatory, and anti-apoptotic is a hot research mechanism of exercise for COPD. Emerging mechanism research focuses typically on exosome and alveolar remodelling studies, and tissue engineering studies. In addition, future studies could delve into the interactions between different mechanisms and attempt to elucidate the recommended motility and intensity of exercise for COPD, particularly in emphysema, idiopathic pulmonary fibrosis, and interstitial lung disease.

## Data Availability

The raw data supporting the conclusions of this article will be made available by the authors, without undue reservation.
